# Quantification of Caval contribution to flow in the Right and Left Pulmonary Artery of Fontan patients with 4D Flow MRI

**DOI:** 10.1186/1532-429X-14-S1-W8

**Published:** 2012-02-01

**Authors:** Pablo Bächler, Natalia Pinochet, Israel Valverde, Sarah Nordmeyer, Titus Kuehne, Gerard Crelier, Cristian Tejos, Pablo Irarrazaval, Sergio Uribe

**Affiliations:** 1 Pontificia Universidad Católica de Chile, Santiago, Chile; 2 King's College London, London, UK; 3 Deutsches Herzzentrum Berlin, Berlin, Germany; 4 ETH and University of Zurich, Zurich, Switzerland

## Background

Knowledge of caval contribution to flow in the Right and Left Pulmonary Artery (RPA and LPA) of Fontan patients is of great interest, because uneven flow distribution to the lungs is associated with pulmonary arteriovenous malformations. However, in Fontan circulation more than 1 source of flow to the RPA and LPA is present, which makes difficult to quantify the flow distribution with standard methods. Although a novel MRI application was developed to quantify caval contribution years ago, it has limitations and it is not applicable to patients with turbulent flow in the Pulmonary Arteries (PA)[1]. We propose a new method to easily quantify the flow distribution in Fontan patients.

## Methods

A 4D Flow sequence was acquired in 12 healthy volunteers and 9 Fontan patients on a 1.5T Philips scan. Flow distribution was quantified with particles traces using the software "GTFlow". The new method consists of emitting particles from a Region Of Interest (ROI) with a temporal resolution of ~40 ms. Then, we quantified the flow distribution by counting the particles arriving to a different ROI. To validate this method, 2 independent observers compared the flow contribution of the main PA to the RPA and LPA in healthy volunteers. This was done calculating net forward flow, which was used to validate the distribution of flow obtained with our new method. Thereafter, we quantified the flow distribution of the Superior and Inferior Vena Cava (SVC and IVC) to the RPA and LPA in Fontan patients. Statistical analysis was performed with paired t-test and Bland Altman plots.

## Results

There was good agreement when calculating flow distribution of the PA to the RPA and LPA using net forward flow and particle traces in volunteers. Mean flow distribution to the RPA was:

53.3%±2.4% with forward volume vs. 53.9%±3.2% with particle traces; p-value=0.53 (Observer 1).

53.4%±3.6% with forward volume vs. 53.9%±3.8% with particle traces; p-value=0.68 (Observer 2).

**Figure 1:**
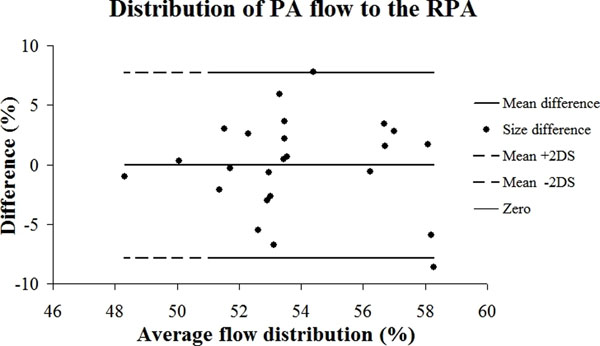
Bland Altman plot representing the mean differences of flow distribution between both methods. (mean difference: 0.04%, range: -8.6 to 7.8%). PA=Pulmonary Artery, RPA=Right Pulmonary Artery.

Figure 1 shows a Bland Altman plot representing the mean differences of flow distribution between both methods.

Variability of measurements between observers was low (Forward volume: mean difference=0.1%, p-value=0.91; Particle traces: mean difference=0.02%, p-value=0.99).

**Figure 2:**
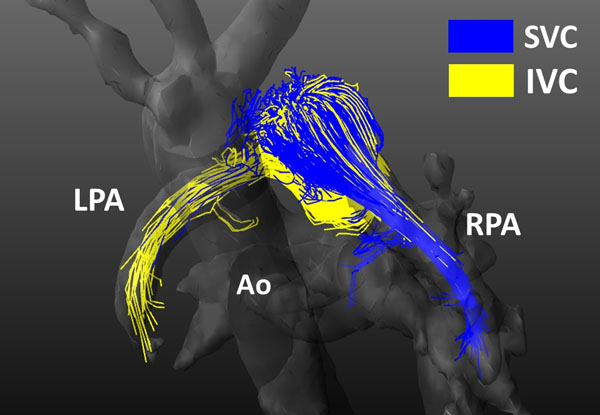
Particle traces showing caval distribution in a Fontan patient at 70% of the cardiac cycle (SVC blood flow distribution: 75% to RPA, 25% to LPA; IVC blood flow distribution: 24% to RPA; 76% to LPA). SVC=Superior Vena Cava, IVC=Inferior Vena Cava, RPA=Right Pulmonary Artery, LPA=Left Pulmonary Artery, Ao=Aorta.

In Fontan patients, the SVC blood flow was mainly directed to the RPA (mean=83%±13%, range=66-99%). Instead, the IVC blood flow was predominantly directed into the LPA, but its distribution was variable among patients (mean=53%±19%, range=21-76%) (Fig. 2).

## Conclusions

We have validated a novel method to calculate the flow distribution when more that 1 vessel contributes with blood flow, such as Fontan patients. This approach revealed that in this group of Fontan patients the SVC blood goes mainly to the RPA, and the IVC blood to the LPA. Quantification of caval flow contribution to the RPA and LPA may identify Fontan patients at risk for developing complications secondary to uneven flow distribution.

## Funding

Fondecyt 11100427 Anillo ACT 079.

## References

[B1] Fogel MA Circulation 1999 99 1215 21 10.1161/01.cir.99.9.1215 10069790

